# Affective touch and attachment style modulate pain: a laser-evoked potentials study

**DOI:** 10.1098/rstb.2016.0009

**Published:** 2016-11-19

**Authors:** Charlotte Krahé, Marianne M. Drabek, Yannis Paloyelis, Aikaterini Fotopoulou

**Affiliations:** 1 Department of Neuroimaging, Institute of Psychiatry, Psychology and Neuroscience, King's College London, London, UK; 2 Arthritis Research UK Pain Centre, University of Nottingham, Nottingham, UK; 3 Research Department of Clinical, Educational and Health Psychology, University College London, London, UK

**Keywords:** affective touch, pain, laser-evoked potentials, attachment style, interoception

## Abstract

Affective touch and cutaneous pain are two sub-modalities of interoception with contrasting affective qualities (pleasantness/unpleasantness) and social meanings (care/harm), yet their direct relationship has not been investigated. In 50 women, taking into account individual attachment styles, we assessed the role of affective touch and particularly the contribution of the C tactile (CT) system in subjective and electrophysiological responses to noxious skin stimulation, namely N1 and N2-P2 laser-evoked potentials. When pleasant, slow (versus fast) velocity touch was administered to the (non-CT-containing) palm of the hand, higher attachment anxiety predicted increased subjective pain ratings, in the same direction as changes in N2 amplitude. By contrast, when pleasant touch was administered to CT-containing skin of the arm, higher attachment anxiety predicted attenuated N1 and N2 amplitudes. Higher attachment avoidance predicted opposite results. Thus, CT-based affective touch can modulate pain in early and late processing stages (N1 and N2 components), with the direction of effects depending on attachment style. Affective touch not involving the CT system seems to affect predominately the conscious perception of pain, possibly reflecting socio-cognitive factors further up the neurocognitive hierarchy. Affective touch may thus convey information about available social resources and gate pain responses depending on individual expectations of social support.

This article is part of the themed issue ‘Interoception beyond homeostasis: affect, cognition and mental health’.

## 1. Introduction

Affective touch and cutaneous pain are two sub-modalities of interoception that have contrasting affective qualities (pleasantness/unpleasantness) and social meanings (care/harm). These modalities are of fundamental homeostatic importance, signalling physiological safety or threat to the organism [1], and are mediated by neurophysiological systems distinct from those serving non-affective tactile afferents [2]. As such, affective, pleasant touch and unpleasant, cutaneous pain have been classified as part of interoception [2], even when the source of the skin stimulation lies outside the body. At a peripheral level, unpleasant (painful) sensations are well characterized in terms of afferent signalling by dedicated nociceptive afferents (C and Aδ fibres). At least some affectively pleasant tactile sensations are also thought to be coded by specialized unmyelinated C tactile (CT) afferent fibres; these are found only in hairy skin (they are absent in non-hairy, i.e. glabrous skin [3]) and microneurography studies have shown that they selectively respond to innocuous tactile stimulation at slow velocities (1–10 cm s^−1^), with their activation being highly correlated with perceived pleasantness [4]. Peripheral pathways coding unpleasant and pleasant sensations project via thalamic pathways to brain regions implicated in interoceptive processing, notably posterior insular cortex, orbitofrontal cortex and anterior cingulate cortex ([1,5,6], see also [7], which distinguishes neural networks for unpleasant and pleasant sensations). Moreover, some of these brain areas have been implicated in the top-down, cognitive modulation of pain and affective touch (for reviews, see [1,6,8]).

However, it is less clear whether these modalities can regulate each other and particularly whether affective touch can influence subjective and neural responses to noxious stimulation. In primates, pro-social, tactile stimulation, mostly licking and grooming behaviours by conspecifics, attenuates neuroendocrine and behavioural responses to stress, with beneficial long-term effects [9,10]. Further, such tactile contact by conspecifics can also activate endogenous analgesic processes mediated by opioid mechanisms [11] and oxytocinergic pathways [12]. The involvement of these neurobiological pathways, implicated in pain regulation (e.g. [13]) as well as the formation and maintenance of close social bonds [14,15], highlights the potential role of social, affective touch for also regulating pain in humans. Indeed, we have recently shown that in humans, intranasal administration of oxytocin can attenuate subjective and neural responses to pain [16]. This is important as oxytocin is thought to be released in response to affective, social touch in mother–infant and adult interactions [17].

To the best of our knowledge, the potential role of affective, social touch on pain has not been systematically studied in humans. Clinical and developmental studies have long suggested that touch-based interventions such as mild or moderate pressure massage and ‘skin-to-skin’ contact can have positive analgesic effects in preterm infants and in adults diagnosed with fibromyalgia and rheumatoid arthritis syndromes (for reviews, see [18,19]). However, such studies have methodological limitations: they cannot account for the mediating mechanisms of the reported effects and even the efficacy of such interventions remains contested [20,21]. Further, studies testing whether concurrent social touch (partner hand-holding) attenuated experimentally induced pain did not involve psychophysical control or measurements regarding touch parameters [22,23].

Despite this scarcity of systematic studies on the modulation of pain by social, affective touch, there are several relevant indications from studies on neighbouring topics. Social support has well-established beneficial effects on a range of physical health outcomes [24]. In experimental studies, social support modulated psychological and neurophysiological responses to stress (e.g. [25]), as well as pain (see [26,27], for reviews). Moreover, in experimental and neuroimaging studies, we have shown that this pain modulation depends on particular ‘embodied’ social support variables (e.g. the presence of another individual), as well as individual differences in the perception of social relationships themselves, namely attachment styles [28–30]. Insecure attachment styles in particular (characterized by negative expectations of social support; [31]), which may be linked with an impoverished oxytocin system [17], seem to moderate the relationship between social support and pain (see also [32]). Higher attachment anxiety (associated with seeking and craving signs of reassurance) led to reduced pain in the presence of a high versus low empathic stranger [30], while higher attachment avoidance (associated with distancing from others and preferring to cope alone) led to increased pain in the presence of a stranger [30] or romantic partner [29].

Accordingly, in this study, we considered the role of individual differences in attachment styles, while examining how subjective and neural responses to noxious stimuli may be modulated by low pressure, slow velocity dynamic touch by another individual, which is expected to evoke pleasant sensations [4]. These investigations afford several methodological advantages compared to hand-holding or ‘massage-like’ manipulations. As aforementioned, this kind of dynamic touch has been associated with neurophysiological specificity at both peripheral and central levels (see [8] for a review). Thus, we can contrast pleasant (slow velocity) touch with fast velocity touch, which is judged to be neutral, i.e. neither pleasant nor unpleasant [33] and which does not optimally activate CT fibres [4]. Moreover, slow, dynamic touch administered to the hairy skin of the arm and to the glabrous (non-hairy) palm of the hand can be perceived as pleasant and communicate social support [34], but only touch to the hairy skin involves CT-afferent signalling [3]. By systematically varying the speed and location of tactile stimulation, we thus tested the effects of pleasant (slow) versus neutral (fast) touch on pain, as well as the separate involvement of bottom-up physiological mechanisms (CT fibre activation in the forearm) from top-down, learned expectations of pleasantness and support in non-CT-containing skin.

Further, a methodological consideration is whether to administer dynamic touch and noxious stimuli in a temporally and spatially synchronous way, or to separate the two modalities in time and body space. Concurrent tactile (non-nociceptive) stimulation can modulate the perception of noxious stimuli by spinal and supraspinal mechanisms (e.g. [35–38]). In such multisensory perception studies, tactile stimuli perceived as emotionally ‘neutral’ can attenuate pain when applied in temporal and spatial congruency (e.g. same dermatomes) with noxious stimuli. However, examining dynamic touch and noxious stimulation in synchrony poses several difficulties: the stimuli and concomitant afferent signalling differ in their temporal properties, and the interplay between activation of C, Aδ and CT fibres has not yet been characterized. Moreover, direct interactions between such modalities during congruent multisensory stimulation may operate primarily at the spinal level [38] and not reflect the regulatory processes of social support targeted in this study, nor the everyday reality of socially supportive tactile interactions which may precede or follow a painful event. Thus, separating the two modalities in time and space is more compatible with the aims of this study. Accordingly, we administered dynamic touch before noxious stimuli and to the opposite side of the body.

To examine the central neural mechanisms underlying the effects of affective touch on pain, we measured laser-evoked potentials (LEPs): deflections in the ongoing electroencephalogram (EEG), reflecting the activation of Aδ fibres in response to transient, noxious, thermal stimulation (brief radiant heat pulses by an infrared laser selectively activating Aδ- and C-fibre skin nociceptors) that can be used to distinguish effects at different stages of nociception and pain processing [16,29,39]. The N1 component (a negative deflection maximal at contralateral temporal electrodes, peaking at approximately 160 ms) mainly reflects very early stages of sensory processing occurring outside conscious awareness, while the later N2 and P2 components (maximal at the scalp vertex, peaking at 200–350 ms) reflect processes underlying the subjective experience of pain [39]. For example, we found that the presence of a romantic partner modulates only the N2–P2 complex [29], congruent with the view that social-cognitive factors modulate pain experience at higher levels of the neurocognitive hierarchy [1].

In sum, we investigated whether different properties of affective, social touch, including bottom-up signals related to the CT afferent system, as well as top-down components related to learned affective and social meanings of touch, may modulate subjective and neural responses to pain in relation to individual differences in attachment anxiety and avoidance. In accordance with previous studies (e.g. [29]), we expected affectively pleasant touch to reduce pain in individuals with higher attachment anxiety and conversely increase it in individuals with higher attachment avoidance relative to neutral (fast velocity) touch. Moreover, we expected the activation of the CT afferent system to modulate earlier stages of pain processing indexed by the N1 component, while pleasant touch not involving the CT system was expected to influence later N2 and P2 components and subjective pain ratings, and thus, at least partly, be linked to higher-order processing.

## 2. Material and methods

### (a) Design

We employed a 2 × 2 mixed design. Stroking velocity (slow: 3 cm s^−1^, versus fast: 18 cm s^−1^, order counter-balanced across participants) was a within-subjects factor, and touch location (CT group: hairy skin of forearm, versus GL group: glabrous skin of palm) was a between-subjects factor. Outcome measures were pain rating and N1, N2 and P2 local peak amplitudes. The moderating effect of attachment styles was examined using continuous scores on the anxiety and avoidance dimensions of a self-report questionnaire.

### (b) Participants

Given gender differences in touch perception [40,41], 50 right-handed women participated in this study. Exclusion criteria were a depression severity score greater than 9 (PHQ-9 questionnaire; [42]), a history of chronic pain, psychiatric, medical or neurological conditions, or having a wound, scar, tattoo or skin irritation/disease on the forearms or hands. One participant was excluded for not following experimental instructions correctly. Participants were randomly assigned to the CT group (*n* = 24) or the GL group (*n* = 25). Mean age was 23.76 (s.d. = 3.76) years and did not significantly differ between groups. Mean body mass index (BMI) differed significantly between groups (CT group: *M* *=* 22.70, s.d. *=* 3.74; GL group: *M* *=* 20.84, s.d. *=* 2.59; *t*_47_ = −2.03, *p* *=* 0.049); thus, BMI was taken into account in the analyses.

### (c) Procedure

After consenting and completing questionnaires, participants were familiarized with the laser equipment, and their individual experimental and distractor laser pulse intensities were determined. We then recorded participants' EEG while administering a baseline nociceptive stimulation block (no touch). Participants then received the two stroking velocity conditions, separated by a 10-min break to minimize carryover effects [43].

In order to reinforce the main stroking velocity manipulation, each stroking velocity condition consisted of four tactile stimulation mini-blocks (all same velocity) alternating with nociceptive mini-blocks, during which EEG was recorded. In each mini-block, participants received brush strokes to the right arm/palm of the hand (depending on touch location group), after which they rated the touch on dimensions of pleasantness, intensity and comfort (manipulation checks), and subsequently received laser stimuli to the dorsum of their left hand. Tactile and nociceptive stimulation therefore occurred in close sequence but were temporally and spatially distinct. After the second stroking velocity condition, participants were fully debriefed and paid for their time (120 min) in this single session study.

### (d) Materials and measures

#### (i) Tactile stimulation

Tactile stimulation was administered by an unfamiliar, trained experimenter, using a cosmetic make-up brush (Natural hair Blush Brush, No 7, The Boots Company). Participants rested their right arm on a table behind a screen (preventing them from seeing the experimenter during tactile stimulation) with their palm facing upwards (as in [41]). Two 9 cm long by 4 cm wide areas were marked contiguously along participants' right volar forearm between wrist and elbow (CT group) or across their right palm (GL group). To ensure a constant pressure, the brush splayed no wider than a 4 cm window. In each stroking velocity condition, touch was administered in four 30-s mini-blocks in an elbow-to-wrist direction [40,43] at slow (3 cm s^−1^—a single brush stoke) or fast (18 cm s^−1^—6 strokes) velocities with 3-s pauses between strokes and alternating between skin areas on consecutive brush strokes to avoid habituation. After each mini-block, participants rated the received touch from −5 (*not at all*) to 5 (*extremely*) pleasant/intense/comfortable. We used mean ratings across mini-blocks to test for differences in the affective quality of the touch across stroking velocity conditions.

#### (ii) Nociceptive stimulation

As in Paloyelis *et al*. [16] and Krahé *et al*. [29], we used an infrared neodymium yttrium aluminium perovskite (Nd:YAP) laser (Electronical Engineering, Italy) with a wavelength of 1340 nm to generate radiant heat pulses. Pulse duration was 4 ms and spot diameter 5 mm at the skin site (dorsal digits of the left hand). Each block contained 40 experimental and 20 distractor pulses, presented in pseudorandom order (see the electronic supplementary materials for details). Participants' self-reported pain was measured on an 11-point scale ranging from 0 (*no pinprick sensation*) to 10 (*worst pinprick sensation imaginable*); it is the ensuing pinprick (first pain) sensation that is generated from Aδ-fibre activation and reflected in LEPs [39]. Mean pain ratings for the experimental pulses in each block (across the four mini-blocks) served as the measure of subjective pain report.

#### (iii) Electroencephalogram recording and laser-evoked potential analysis

As in Krahé *et al*. [29], EEG data were recorded using a 16-channel Guger Technologies Medical Engineering GmbH (g.tec; Austria) elasticized cap with an active electrode system and g.tec g.recorder software. Data were collected from 11 electrodes positioned along the midline (Fz, FCz, Cz, CPz, Pz) and temporal regions (T7, C5, C3, T8, C6, C4) according to the international 10–20 system. An electrode on the right earlobe was used as the recording reference, and electrodes on the nose and bilateral mastoids were included for offline re-referencing (see the electronic supplementary materials for details). The local peak-to-baseline amplitude of N2 (most negative peak 0–350 ms from stimulus onset) and P2 (most positive peak 0–600 ms) components was measured at the Cz electrode (referenced to averaged bilateral mastoid electrodes), and that of the N1 component (most negative peak 0–270 ms) was measured at the C6 electrode (contralateral to the stimulated hand), using the Fz electrode as reference [16,29]. Data exclusion due to technical issues resulted in a final sample of *N* *=* 43 for N1 and *N* *=* 41 for N2/P2 analyses (see the electronic supplementary materials). Missing data were not systematically associated with any condition and were estimated using a maximum likelihood with missing values procedure (see Statistical analyses).

#### (iv) Adult attachment style

We used the 36-item Experiences in Close Relationships Revised (ECR-R; [44]) questionnaire to measure the attachment anxiety and attachment avoidance dimensions. This questionnaire is well validated [45] and demonstrates excellent internal consistency; Cronbach's *α* = 0.92 (attachment anxiety) and *α* = 0.91 (attachment avoidance) in the present sample.

### (e) Statistical analyses

Statistical analyses were carried out in Stata 13 [46]. To test our hypotheses, we estimated multi-group models with maximum likelihood with missing values estimation using the ‘sem’ and ‘mlmv’ commands. The difference between touch velocities was considered by calculating difference scores (fast velocity minus slow velocity; as in e.g. [33]) for each outcome variable. In each analysis, the grouping variable was touch location (CT versus GL group) and predictors were attachment anxiety, attachment avoidance and their interaction. We controlled for baseline differences in outcome variables, as well as demographic variables, by including them as covariates in the corresponding analyses. All continuous predictors were mean-centred [47]. We ran *χ*^2^ (Wald) tests (‘estat ginvariant’ command) to examine which parameters differed significantly between touch location groups [48]. To visualize effects of attachment style, we plotted effects at ±1 s.d. of the sample mean for attachment anxiety and avoidance, using unstandardized parameter estimates [47].

## 3. Results

### (a) Descriptive statistics

Table 1 presents descriptive statistics. A mixed ANOVA with stroking velocity as repeated-measures variable and touch location group as between-subject variable confirmed that the slow stroking velocity was rated as more pleasant than the fast stroking velocity across touch locations groups, supporting the predicted distinction in the affective quality between the two stroking velocities but not between touch locations. This pattern of results was similar for intensity and comfort ratings (see the electronic supplementary material, table S1 for descriptive and ANOVA results).

**Table 1.** Descriptive statistics (mean and s.d.) for attachment style dimensions and pain-related outcome measures.

	stimulation block	touch location group	
		CT (hairy skin of forearm)	GL (glabrous palm of the hand)
attachment anxiety (scale 1–7)	n.a.	2.88 (0.94)	3.02 (1.10)
attachment avoidance (scale 1–7)	n.a.	2.91 (0.92)	3.08 (0.93)
pain rating (scale 0–10)	baseline	4.09 (1.34)	4.29 (1.53)
	slow velocity touch	4.15 (1.40)	4.19 (1.47)
	fast velocity touch	4.44 (1.36)	4.17 (1.68)
N1 local peak amplitude (µV)	baseline	−9.12 (5.48)	−9.28 (6.10)
	slow velocity touch	−6.83 (3.74)	−8.06 (4.69)
	fast velocity touch	−5.75 (3.09)	−7.23 (3.55)
N2 local peak amplitude (µV)	baseline	−12.65 (8.25)	−18.39 (11.59)
	slow velocity touch	−11.76 (7.68)	−14.26 (11.89)
	fast velocity touch	−10.61 (7.43)	−14.51 (9.88)
P2 local peak amplitude (µV)	baseline	21.16 (11.77)	25.56 (8.19)
	slow velocity touch	19.14 (9.77)	22.75 (8.29)
	fast velocity touch	19.41 (9.57)	22.53 (6.66)

### (b) Pain rating

**Figure 1.**
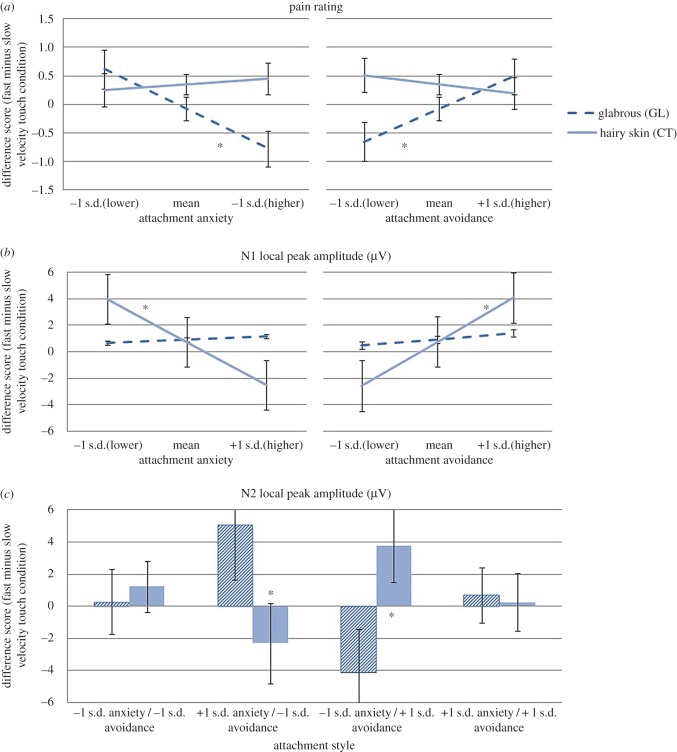
Effects of attachment anxiety and attachment avoidance on difference scores (fast minus slow velocity touch condition) for (*a*) pain rating (plotted at low (−1 s.d.), mean and high (+1 s.d.) attachment scores), (*b*) N1 local peak amplitude (plotted at low (−1 s.d.), mean and high (+1 s.d.) attachment scores) and (*c*) effects of attachment anxiety by attachment avoidance on N2 local peak amplitude (plotted at low (−1 s.d.) and high (+1 s.d.) attachment anxiety and attachment avoidance scores) for GL (glabrous skin) and CT (hairy skin) touch location groups. Error bars denote ±1 s.e. from the mean. Note: we oriented ourselves on the negative-going N1 and N2 components and subtracted the slow velocity from the fast velocity touch condition so that negative difference scores indicate attenuated neural responses to slow versus fast velocity touch for N1 and N2; for pain rating, greater pain is reflected in more positive values, and so a negative difference score denotes greater pain for slow versus fast velocity touch.

Higher attachment anxiety predicted increased subjective pain rating in response to slow versus fast touch in the GL group (*b* = −0.68, s.e. = 0.23, *p* = 0.004, 95% CIs [−1.14; −0.22]) but not the CT group (*b* = 0.10, s.e. = 0.20, *p* = 0.616, 95% CIs [−0.29; 0.49]); *p* = 0.011 for the parameter difference across groups (figure 1*a*). Conversely, higher attachment avoidance predicted attenuated pain rating in response to slow versus fast touch in the GL group (*b* = 0.62, s.e. = 0.28, *p* = 0.027, 95% CIs [0.07; 1.17]) but not the CT group (*b* = −0.17, s.e. = 0.24, *p* = 0.465, 95% CIs [−0.64; 0.29]); *p* = 0.031 for parameter difference across groups (figure 1*a*). Effects were not due to an interaction between attachment dimensions in either touch location group (GL group: *b* = −0.16, s.e. = 0.14, *p* = 0.256, 95% CIs [−0.42; 0.11]; CT group: *b* = 0.38, s.e. = 0.27, *p* = 0.163, 95% CIs [−0.16; 0.92]). Thus, when pleasant, slow (versus fast) touch was administered to the palm of the hand, higher attachment anxiety predicted an increase and higher attachment avoidance predicted a decrease in pain ratings.

### (c) Laser-evoked potential results

#### (i) N1 local peak amplitude

In contrast to the pain rating findings, higher attachment anxiety predicted an attenuated N1 response to slow versus fast touch in the CT group (*b* = −3.13, s.e. = 1.07, *p* = 0.004, 95% CIs [−5.23; −1.03]) but not the GL group (*b* = 0.24, s.e. = 0.43, *p* = 0.574, 95% CIs [−0.60; 1.01]); *p* = 0.004 for parameter difference across groups (figure 1*b*). Conversely to attachment anxiety, higher attachment avoidance predicted an augmented N1 response to slow versus fast touch in the CT group (*b* = 3.48, s.e. = 1.05, *p* = 0.001, 95% CIs [1.43; 5.54]) but not the GL group (*b* = 0.49, s.e. = 0.50, *p* = 0.332, 95% CIs [−0.50; 1.47]); *p* *=* 0.001 for the parameter difference across groups (figure 1*b*). Effects were not due to an interaction between attachment dimensions in either touch location group (CT: *b* = −1.77, s.e. = 1.39, *p* = 0.203, 95% CIs [0.20; −4.49]; GL: *b* = −0.34, s.e. = 0.25, *p* = 0.164, 95% CIs [0.164; −0.82]). In brief, when pleasant, slow (versus fast) touch was administered to the CT-containing hairy skin of the arm, higher attachment anxiety predicted an attenuated N1 local peak amplitude, while higher attachment avoidance predicted an enhanced effect.

#### (ii) N2 local peak amplitude

There was no effect of attachment anxiety (CT group: *b* = −1.72, s.e. = 1.15, *p* = 0.135, 95% CIs [−3.97; 0.53]; GL group: *b* = 2.32, s.e. = 1.37, *p* = 0.090, 95% CIs [−0.36; 5.00]) or attachment avoidance (CT group: *b* = −1.33, s.e. = 1.40, *p* = 0.340, 95% CIs [−1.40; 4.07]; GL group: *b* = −2.25, s.e. = 1.65, *p* = 0.173, 95% CIs [−5.48; 0.98]). However, the attachment anxiety by attachment avoidance interaction was significant in the CT group (*b* = −3.29, s.e. = 1.55, *p* = 0.034, 95% CIs [−6.32; −0.24]) but not the GL group (*b* = 0.72, s.e. = 0.77, *p* = 0.352, 95% CIs [−0.79; 2.23]); *p* = 0.021 for the parameter difference across groups. Thus, when pleasant, slow (versus fast) touch was administered to the CT-containing hairy skin of the arm, higher attachment anxiety predicted an attenuated N2 response only when attachment avoidance was lower, while higher attachment avoidance predicted an augmented N2 response only when attachment anxiety was lower; opposite (but non-significant) effects were observed when touch was applied to the palm of the hand (figure 1*c*).

#### (iii) P2 local peak amplitude

Neither attachment anxiety or avoidance, nor their interaction predicted P2 response to slow versus fast touch in either group (tests for GL versus CT group parameter differences: *p* = 0.375 for attachment anxiety; *p* = 0.489 for attachment avoidance; *p* = 0.389 for the anxiety by avoidance interaction).

### (d) Association between pleasantness ratings and pain modulation

The degree to which participants perceived slow and fast velocity touch to be pleasant was not associated with pain modulation in the corresponding conditions (see the electronic supplementary material, table S2).

## 4. Discussion

This study investigated whether affective touch modulated subjective and neural responses to pain and whether the direction of effects depended on individual differences in attachment styles. In line with previous studies [29,30], we expected that affectively pleasant touch would reduce pain-related outcomes in individuals with higher attachment anxiety and conversely increase them in individuals with higher attachment avoidance. We further predicted that CT afferent signalling would drive modulation of early stages of pain-related processing (N1), but would not be critical for effects at later, higher-order stages of processing (N2–P2 and pain ratings).

In line with our predictions, higher attachment anxiety was associated with an attenuating effect of slow (versus fast) touch on the N1 amplitude, while higher attachment avoidance predicted an enhancing effect on the N1 amplitude. These results are similar to our previous findings that higher attachment anxiety was related to attenuation of pain-related outcomes when interactions signalled a socially supportive intent (e.g. high empathy), while social interactions *per se* (i.e. the mere presence of others) increased pain in individuals higher in attachment avoidance [29,30]. Higher attachment anxiety is linked to craving closeness and reassurance from others: affectively pleasant, slow velocity touch may promote representations of affiliation and bonding, enhancing perceived support and attenuating early processing of noxious stimuli (see also [27]). On the other hand, higher attachment avoidance is linked to avoiding closeness and preferring to cope alone; here, touch may promote negative representations of the unavailability of others, maintaining perceived threat and enhancing early processing of noxious stimuli (see also [27]). Thus, as expected, the effects of affective touch on the earliest stage of pain-related neural processing depended on attachment style and appeared to be mediated by the CT afferent system, as effects on N1 were found only in the group that received touch to the CT-fibre-abundant hairy skin of the arm.

Although the N1 component can be modulated by both noxious stimulation and vicarious pain [49], theories of interoception have posited that social-cognitive factors are mostly integrated in areas, such as the anterior cingulate cortex and anterior insula, which are captured by the later N2–P2 complex [1,39]. Thus, we expected this complex to be influenced by the perceived pleasantness of slow (versus fast) touch in both touch locations. Indeed, stroking the palm of the hand (which does not contain CT fibres) at slow versus fast velocities can also give rise to feelings of pleasantness, possibly due to alternative, not as yet understood, bottom-up mechanisms, as well as learned expectations of pleasantness [41,43]. This was supported by our manipulation checks, which showed that slow velocity touch was rated as more pleasant than fast velocity touch at both touch locations. Our N2 findings were broadly consistent with these hypotheses, although some particular effects were unexpected. Specifically, the N2 results mirrored our N1 findings in the group receiving touch to the hairy skin of the arm, with the additional qualification that effects of higher attachment anxiety were seen only when attachment avoidance was lower, and effects of higher attachment avoidance only when anxiety was lower. These results confirm that attachment styles influence how affective touch modulates pain-related responses at higher levels of the neurocognitive hierarchy where both CT-based, bottom-up signals and learned affective and social meanings of touch may be relevant. Nevertheless, it is noteworthy that in the group that received touch to CT-containing skin, these N2 results were not mirrored by findings regarding subjective ratings. In addition, we did not find any moderating effects of attachment styles on the relationship between affective touch and P2 amplitude. We found a similar lack of P2 results in a styles examining the anti-nociceptive effects of oxytocin [16]. While LEPs in general have been conceptualized to reflect neural responses to salient bodily threats in the environment [50], the P2 component is specifically implicated in reflecting factors signalling stimulus salience (e.g. [51]). Thus, affective touch may not specifically modulate the salience of noxious stimuli in the context of individual differences in attachment style, although future research is needed in this regard.

By contrast, pleasant, slow (versus fast) touch influenced subjective ratings in the group receiving touch to the palm of the hand, with higher attachment anxiety being associated with increased ratings, while higher attachment avoidance predicted decreased ratings. N2 responses showed the same direction of effects in this group but these effects were not significant. It is possible that the social ambiguity involved in slow, pleasant stroking of the palm drove these results, with individuals with higher attachment anxiety being particularly preoccupied by its unclear meaning, while individuals with higher avoidance preferred this less ‘intimate’ form of social support. This interpretation remains speculative, however, and future studies should further explore the perceived social intentions of various types of affective touch to different body parts [34].

We selected 3 cm s^−1^ as the slow stroking velocity and 18 cm s^−1^ as the fast stroking velocity on the basis that 3 cm s^−1^ lies within the optimal range for activating the CT system, and dynamic touch at this velocity is perceived as pleasant [4], while 18 cm s^−1^ is not within this optimal velocity range and is perceived as significantly less pleasant than CT-optimal touch [33]. Further, perceived pleasantness does not differ between 18 cm s^−1^ and faster velocity touch at 27 cm s^−1^ [52]; the latter is similar to 30 cm s^−1^, a velocity which does not preferentially activate the CT system [4]. Moreover, by using difference scores, we investigated effects on the relative difference between slow and fast stroking velocity: therefore, we could not explore whether effects were confined to slow velocity touch but rather saw that effects here were greater than for fast velocity touch. Here, it should also be noted that the degree to which participants perceived slow and fast velocity touch to be pleasant was not related to pain modulation in the corresponding conditions, although as reported, the two velocities differed in perceived pleasantness as intended. Therefore, it seems to be the distinction in pleasantness between the two touch velocities rather than the variations in pleasantness within these two touch velocity conditions that is important for our observed effects.

Taken together, our N2 and pain report findings suggest that CT pathways may contribute to the late processing of noxious stimuli, but unlike in the case of our N1 findings and early processing of noxious stimuli where CT afferent signalling appeared to drive our effects, the relationships between the CT afferent system, learned affective and social meanings of touch and conscious perception of pain remain unclear and require further investigation.

We have argued that the ability of affective slow dynamic touch to signal and promote a caring and socially supportive orientation from others [34] suggests that it may be conceptualized as a form of embodied social support. In carefully varying and controlling physiological parameters of touch and examining specifically the contribution of CT fibres, we showed that effects of affectively pleasant touch on pain depend on individual differences in attachment styles. While affective, social touch as well as social support more generally have beneficial effects on a range of health outcomes [18,19,24], we can add that—as with other forms of social support [29]—embodied support in the form of pleasant, social touch is not unequivocally pain-attenuating, but rather its effects depend on individual differences in how social support from others and social relationships more generally are perceived. Thus, future studies should investigate further individual-difference variables such as gender (we only tested women in the current study) and genetic variations in the mu-opioid receptor gene. Animal studies have shown a relationship between the mu-opioid receptor gene and attachment behaviour [53], and different attachment behaviours are exhibited depending on different polymorphisms of the gene [54]. In humans, certain alleles have been linked to a fearful attachment style, characterized by high attachment anxiety and avoidance [55], indicating that variations in this gene may be associated with individual differences in attachment styles. Moreover, the social context of touch (whether administered by a romantic partner, a stranger or a machine) requires further study to tease apart possible social versus non-social elements in the effects of touch. Although we have not tested this factor directly, we believe that the key role of individual differences in attachment styles—a trait which is inextricably linked to social processes—in moderating the effects of affective touch on pain in this study warrants further research into the specific role of social touch in pain. Related to this, two studies contrasting social and non-social manipulations [22,56] found that social conditions reduced pain to a greater extent than did non-social conditions. In addition, we investigated effects on pain which was experimentally induced in healthy volunteers, but examining the perception of affective touch and its effects on pain modulation in individuals with chronic pain is also an important avenue of research (see [57] for differences in the perception of slow versus fast velocity touch in healthy volunteers versus fibromyalgia patients). We separated affective touch and pain in time and body space. Characterizing the interactive effects of C, Aδ and CT fibres remains an important aim for studies into the multisensory integration of these two interoceptive modalities. Lastly, we focused on the receipt of affective, social touch, but evidence suggests that providing touch may also have beneficial effects [52], and thus the reciprocal nature of social interactions should be taken into account in future studies.

In sum, we found that effects of affective touch on subjective and neural responses to pain depend on individual differences in attachment style. As expected, the activation of the CT afferent system affected pain at earlier stages of pain processing (N1), while it did not appear to have an equally clear role at higher levels of pain processing as measured by the N2 and P2 components and subjective pain ratings.

## Supplementary Material

Supplementary information on nociceptive stimulation, EEG recording and LEP analysis

## Supplementary Material

Supplementary Tables

## Supplementary Material

Dataset

## Data accessibility

The article's supporting data have been uploaded as the electronic supplementary materials.
