# Supplementary material for: Affective touch and attachment style modulate pain: a laser-evoked potentials study
Source: Philos Trans R Soc Lond B Biol Sci. 2016 Nov 19;371(1708):20160009. doi: 10.1098/rstb.2016.0009 (PMC5062098; doi:10.1098/rstb.2016.0009)
Supplement: Supplementary information on nociceptive stimulation, EEG recording and LEP analysis [file rstb20160009supp1.pdf]

Supplementary material for Krahé C, Drabek M M, Paloyelis Y, Fotopoulou A. 2016 Affective touch and attachment style modulate pain: a laser-evoked potentials study. Phil. Trans. R. Soc. B 20160009. http://dx.doi.org/10.1098/rstb.2016.0009

Affective touch and attachment style modulate pain: A laser-evoked potentials study

Supplementary information on nociceptive stimulation, EEG recording and LEP analysis

Nociceptive stimulation. As in Paloyelis, Krahé [1] and Krahé, Paloyelis [2]), we used an infrared neodymium yttrium aluminium perovskite (Nd:YAP) laser (Electronical Engineering, Italy) with a wavelength of 1034nm to generate radiant heat pulses. Pulse duration was 4 ms and spot diameter 5mm at the skin site (dorsal digits of the left hand). Spot location was changed after each pulse to avoid nociceptor fatigue and sensitisation. Participants' hand temperature was maintained at a constant level during the laser stimulation. Each block contained 40 experimental and 20 distractor pulses, presented in pseudorandom order. Experimental and distractor pulse intensities were individually adjusted for each participant at the beginning of the experimental session to correspond to a clear pinprick sensation of moderate intensity (M = 3.67J, SD = .52) or a sensation at the threshold of perception (M = 2.06J, SD = .48), respectively. Distractor stimuli and jittered inter-stimulus interval were used to increase the unpredictability of the intensity and timing of the pulses; trial length varied from 10-14 seconds. At baseline (preceding the stroking velocity manipulation), participants received all pulses in one continuous block. During the stroking velocity manipulation, pulses were divided into four mini-blocks consisting of 10 experimental and 5 distractor pulses. Participants' self-reported pain was measured on an 11-point scale ranging from 0 (no pinprick sensation) to 10 (worst pinprick sensation imaginable); it is the ensuing pinprick (first pain) sensation that is generated from Aδ-fibre activation and reflected in LEPs ([3]). Mean pain ratings for the experimental pulses in each block (across the four mini-blocks) served as the measure of subjective pain report.

EEG recording and LEP analysis. EEG data was recorded using a 16-channel Guger Technologies Medical Engineering GmbH (g.tec; Austria) elasticized cap with an active electrode system and g.tec g.recorder software. Data were collected from 11 electrodes positioned along the midline (Fz, FCz, Cz, CPz, Pz) and temporal regions (T7, C5, C3, T8, C6, C4) according to the international 10-20 system. An electrode on the right earlobe was used as the recording reference, and electrodes on the nose and bilateral mastoids were included for offline re-referencing. Eye blink artefacts were assessed by recording the electrooculogram from electrodes above and below the right eye. Data were sampled at 512Hz, applying a notch filter at 50Hz to eliminate power line noise and online filtering between 0.1 and 100Hz. EEG data was processed using the open source toolboxes EEGLAB ([4]) and ERPLAB ([5]) for MATLAB ([6]). As in Krahé, Paloyelis [2], the data was downsampled to 256Hz, offline bandpass filtered between 0.4 – 30Hz, segmented into -200 to 800ms epochs in relation to stimulus onset, and baseline-corrected using the 200ms window before stimulus onset. Trials with muscle and eye blink artefacts were rejected by visual inspection. The local peak-to-baseline amplitude of N2 (most negative peak 0–350ms from stimulus onset) and P2 (most positive peak 0–600ms) components was measured at the Cz electrode using the averaged bilateral mastoid electrodes as reference, and that of the N1 component (most negative peak 0–270 ms) was measured at the C6 electrode (contralateral to the stimulated hand), using the Fz electrode as reference ([1]; [2]). Data exclusion due to technical issues (n =2 EEG recording fault; n =4 N2/P2 mastoid reference fault; n =3 fewer than 26 trials remaining after artefact rejection; n = 3 plausible LEPs could not be detected in any condition) resulted in a final sample N =43 for N1 and N =41 for N2/P2 analyses. Missing data were not systematically associated with any condition and were estimated using a maximum likelihood with missing values procedure (see Statistical analyses in main article).

© The Authors under the terms of the Creative Commons Attribution License http://creativecommons.org/licenses/by/3.0/, which permits unrestricted use, provided the original author and source are credited.

Supplementary material for Krahé C, Drabek M M, Paloyelis Y, Fotopoulou A. 2016 Affective touch and attachment style modulate pain: a laser-evoked potentials study. Phil. Trans. R. Soc. B 20160009. http://dx.doi.org/10.1098/rstb.2016.0009

References

[1] Paloyelis, Y., Krahé, C., Maltezos, S., Williams, S.C., Howard, M.A. & Fotopoulou, A. 2015 The Analgesic Effect Of Oxytocin In Humans: A Double-Blinded Placebo Controlled Cross-Over Study Using Laser-Evoked Potentials. *Journal of Neuroendocrinology*. (doi:10.1111/jne.12347).
[2] Krahé, C., Paloyelis, Y., Condon, H., Jenkinson, P.M., Williams, S.C.R. & Fotopoulou, A. 2015 Attachment style moderates partner presence effects on pain: a laser-evoked potentials study. *Social Cognitive and Affective Neuroscience* **10**, 1030-1037. (doi:10.1093/scan/nsu156).
[3] Lee, M.C., Mouraux, A. & Iannetti, G.D. 2009 Characterizing the cortical activity through which pain emerges from nociception. *The Journal of Neuroscience* **29**, 7909-7916. (doi:10.1523/jneurosci.0014-09.2009).
[4] Delorme, A. & Makeig, S. 2004 EEGLAB: an open source toolbox for analysis of single-trial EEG dynamics including independent component analysis. *Journal of neuroscience methods* **134**, 9-21.
[5] Lopez-Calderon, J. & Luck, S.J. 2014 ERPLAB: An Open-Source Toolbox for the Analysis of Event-Related Potentials. *Frontiers in Human Neuroscience* **8**. (doi:10.3389/fnhum.2014.00213).
[6] R2011a *MATLAB and Statistics Toolbox Release* Natick, Massachusetts, United States, The MathWorks, Inc.

© The Authors under the terms of the Creative Commons Attribution License http://creativecommons.org/licenses/by/3.0/, which permits unrestricted use, provided the original author and source are credited.
