# Supplementary material for: Affective touch and attachment style modulate pain: a laser-evoked potentials study
Source: Philos Trans R Soc Lond B Biol Sci. 2016 Nov 19;371(1708):20160009. doi: 10.1098/rstb.2016.0009 (PMC5062098; doi:10.1098/rstb.2016.0009)
Supplement: Supplementary Tables [file rstb20160009supp2.pdf]

Supplementary material for Krahé C, Drabek M M, Paloyelis Y, Fotopoulou A. 2016 Affective touch and attachment style modulate pain: a laser-evoked potentials study. Phil. Trans. R. Soc. B 20160009. http://dx.doi.org/10.1098/rstb.2016.0009

Supplementary Table 1: *Manipulation checks – descriptive and ANOVA results*

	Mean (SD)				ANOVA results		
	Condition	GL	CT	Total	Stroking velocity	Touch location group	Stroking velocity x Touch location group
Pleasantness rating	Slow velocity	2.91 (1.45)	2.66 (1.65)	**2.79** (1.54)	**F(1, 47) = 20.65, p < .001**	F(1, 47) = .10, p = .750	F(1, 47) = .47, p = .500
	Fast velocity	1.88 (1.21)	1.90 (1.53)	**1.89** (1.36)			
	Total	2.40 (1.42)	2.28 (1.62)				
Intensity rating	Slow velocity	-1.75 (2.80)	-0.56 (2.63)	**-1.17** (2.76)	**F(1, 47) = 17.44, p < .001**	F(1, 47) = 2.09, p = .155	F(1, 47) = 0.35, p = .555
	Fast velocity	-0.39 (2.64)	0.46 (2.55)	**0.03** (2.61)			
	Total	-1.07 (2.78)	-0.05 (2.62)				
Comfort rating	Slow velocity	2.96 (2.07)	3.11 (1.62)	**3.04** (1.84)	**F(1, 47) = 17.72, p < .001**	F(1, 47) = .13, p = .722	F(1, 47) = .01, p = .907
	Fast velocity	2.09 (2.06)	2.29 (1.70)	**2.19** (1.88)			
	Total	2.53 (2.09)	2.70 (1.70)				

© The Authors under the terms of the Creative Commons Attribution License http://creativecommons.org/licenses/by/3.0/, which permits unrestricted use, provided the original author and source are credited.

Supplementary material for Krahé C, Drabek M M, Paloyelis Y, Fotopoulou A. 2016 Affective touch and attachment style modulate pain: a laser-evoked potentials study. Phil. Trans. R. Soc. B 20160009. http://dx.doi.org/10.1098/rstb.2016.0009

Supplementary Table 2. *Touch pleasantness ratings and pain-related outcomes – regression results*

**Outcome**	**Stroking velocity condition**	**Predictors**	***b* coefficient**	***SE***	***p* value**	**[95% Conf.**	
Pain rating	Slow velocity	Pleasantness rating slow velocity touch	0.06	0.14	.680	-0.22	0.33
		Touch location group	-0.05	0.42	.915	-0.89	0.80
	Fast velocity	Pleasantness rating fast velocity touch	-0.16	0.16	.345	-0.49	0.17
		Touch location group	0.30	0.44	.498	-0.59	1.19
N1	Slow velocity	Pleasantness rating slow velocity touch	-0.12	0.46	.799	-1.05	0.82
		Touch location group	1.20	1.46	.417	-1.76	4.15
	Fast velocity	Pleasantness rating fast velocity touch	0.09	0.40	.817	-0.71	0.89
		Touch location group	1.49	1.04	.163	-0.63	3.60
N2	Slow velocity	Pleasantness rating slow velocity touch	0.86	1.15	.457	-1.47	3.20
		Touch location group	2.87	3.41	.406	-4.07	9.81
	Fast velocity	Pleasantness rating fast velocity touch	-0.40	1.01	.697	-2.44	1.65
		Touch location group	3.88	2.77	.170	-1.73	9.49
P2	Slow velocity	Pleasantness rating slow velocity touch	0.47	1.02	.648	-1.61	2.56
		Touch location group	-3.41	3.07	.275	.9.67	2.84
	Fast velocity	Pleasantness rating fast velocity touch	-0.08	0.97	.936	-2.04	1.88
		Touch location group	-3.13	2.67	.248	-8.55	2.28

© The Authors under the terms of the Creative Commons Attribution License http://creativecommons.org/licenses/by/3.0/, which permits unrestricted use, provided the original author and source are credited.
