# Supplementary material for: Affective touch and attachment style modulate pain: a laser-evoked potentials study
Source: Philos Trans R Soc Lond B Biol Sci. 2016 Nov 19;371(1708):20160009. doi: 10.1098/rstb.2016.0009 (PMC5062098; doi:10.1098/rstb.2016.0009)
Supplement: Dataset [file rstb20160009supp3.pdf]

Krahé C, Drabek M M, Paloyelis Y, Fotopoulou A. 2016 Affective touch and attachment style modulate pain: a laser-evoked potentials study. Phil. Trans. R. Soc. B 20160009. http://dx.doi.org/10.1098/rstb.2016.0009

id	group	order	age	bmi	PHQ9_score	ECR_anxiety	ECR_avoida	mean_toucl	mean_toucl	mean_toucl	mean_toucl	mean_toucl	mean_toucl	mpain_base	mpain_slow	mpain_fast
1.00	GL	slowvelo-fa:	23.62	17.72	4.00	1.39	1.44	4.50	-1.00	5.00	-0.75	3.25	-0.50	5.03	3.13	5.18
2.00	GL	fastvelo-slo	24.08	18.19	0.00	2.44	3.44	3.75	-1.00	3.75	2.50	1.00	2.50	4.30	4.65	5.13
3.00	CT	slowvelo-fa:	22.20	22.62	8.00	3.00	2.89	1.75	0.50	2.25	1.25	1.00	0.50	2.20	2.98	2.20
4.00	GL	fastvelo-slo	28.21	19.22	3.00	2.11	2.67	4.50	2.25	4.00	0.75	2.25	0.25	5.83	5.48	5.73
5.00	CT	slowvelo-fa:	29.40	29.05	0.00	1.72	2.44	5.00	2.50	4.75	4.25	2.25	4.25	3.05	3.08	2.75
6.00	CT	fastvelo-slo	23.98	27.89	7.00	1.78	3.00	-0.50	-0.50	0.00	0.00	0.00	0.00	5.50	6.98	6.75
7.00	CT	fastvelo-slo	23.02	34.48	1.00	3.89	2.11	1.75	-1.25	4.75	1.75	1.00	5.00	3.65	3.18	5.40
8.00	GL	fastvelo-slo	19.64	20.37	0.00	4.33	3.50	1.75	0.75	1.75	1.00	-1.00	1.00	5.23	5.35	5.25
9.00	GL	fastvelo-slo	31.60	19.82	0.00	1.78	2.83	1.50	2.00	2.00	1.25	2.25	1.50	4.49	4.73	3.74
10.00	GL	slowvelo-fa:	22.48	17.90	2.00	3.94	3.11	4.75	-5.00	5.00	3.75	-4.75	4.50	8.45	7.58	7.93
11.00	GL	fastvelo-slo	19.44	20.20	0.00	1.17	2.06	4.00	-0.75	5.00	2.50	0.00	5.00	3.78	3.85	2.67
12.00	CT	slowvelo-fa:	26.18	20.48	0.00	2.44	2.44	2.50	2.00	3.75	-0.75	2.50	2.75	5.95	4.98	4.78
13.00	CT	fastvelo-slo	31.25	18.37	3.00	3.89	1.94	5.00	-5.00	5.00	4.00	-4.25	4.75	5.82	4.50	3.73
14.00	CT	fastvelo-slo	21.12	22.29	0.00	2.56	2.61	4.50	1.00	4.75	2.00	1.50	3.00	3.83	4.16	4.05
15.00	CT	slowvelo-fa:	22.46	23.71	3.00	3.44	4.44	4.00	-1.00	3.75	2.75	0.50	3.00	5.03	5.32	4.88
16.00	GL	slowvelo-fa:	23.03	19.11	1.00	3.06	3.17	2.25	-3.75	2.50	2.50	-1.50	2.50	3.15	3.45	3.03
17.00	GL	fastvelo-slo	30.61	20.86	3.00	2.56	4.28	3.75	1.50	3.00	2.00	-1.00	1.50	4.31	4.15	4.25
18.00	GL	fastvelo-slo	21.52	24.28	2.00	4.22	3.44	0.75	1.75	-5.00	0.50	2.50	-5.00	4.08	5.28	3.98
19.00	GL	fastvelo-slo	21.10	20.00	1.00	5.17	5.50	1.50	-4.25	4.50	2.00	-5.00	5.00	4.55	5.35	4.38
20.00	GL	fastvelo-slo	23.23	21.48	1.00	3.28	3.28	1.00	-5.00	3.25	0.00	-4.75	3.00	1.92	1.58	2.03
21.00	CT	fastvelo-slo	30.72	23.53	0.00	3.94	2.61	3.75	-2.75	3.75	3.25	2.75	3.00	4.35	4.23	3.85
22.00	GL	fastvelo-slo	22.57	22.35	0.00	3.17	2.89	4.00	-4.50	4.25	3.00	-1.50	2.75	3.97	3.45	2.75
23.00	CT	slowvelo-fa:	21.85	20.94	2.00	2.17	2.28	4.00	-5.00	2.25	3.50	-2.75	0.75	4.20	4.50	5.68
24.00	GL	slowvelo-fa:	22.04	21.08	1.00	4.29	4.22	1.25	0.50	2.00	0.00	1.00	0.50	4.80	5.36	6.15
25.00	CT	fastvelo-slo	22.03	19.81	0.00	3.50	2.78	-0.50	0.00	0.00	1.50	-0.25	2.25	2.64	2.80	3.33
26.00	GL	slowvelo-fa:	23.38	28.31	1.00	4.41	3.89	1.50	-0.50	1.50	1.00	0.50	2.25	3.69	3.73	2.53
27.00	GL	slowvelo-fa:	21.53	18.36	0.00	1.33	1.00	4.50	3.25	5.00	2.75	2.75	3.25	4.23	5.15	5.55
28.00	GL	slowvelo-fa:	23.90	18.96	5.00	3.17	1.89	3.75	-4.50	3.75	3.00	3.00	3.50	3.08	2.44	2.55
29.00	CT	slowvelo-fa:	20.79	23.00	6.00	2.06	3.06	3.25	2.00	3.50	1.50	2.75	1.75	3.95	3.70	3.68
30.00	CT	fastvelo-slo	23.97	19.81	0.00	2.78	2.83	0.50	-5.00	1.00	1.25	-4.50	2.00	2.33	2.15	3.48
31.00	CT	fastvelo-slo	24.10	21.30	1.00	2.94	2.11	4.25	4.25	4.25	2.50	2.50	2.25	3.65	3.67	3.25
32.00	GL	slowvelo-fa:	21.61	22.48	2.00	3.50	3.50	4.50	-3.75	3.50	1.75	-3.50	2.00	5.80	4.85	5.95
33.00	CT	fastvelo-slo	19.18	23.44	0.00	4.39	1.17	4.75	-2.75	5.00	4.00	3.25	4.50	4.30	4.50	4.60
34.00	CT	slowvelo-fa:	30.88	21.62	4.00	3.82	3.39	4.25	2.25	3.50	2.75	3.25	3.00	4.35	2.68	2.20
35.00	GL	slowvelo-fa:	28.58	25.59	0.00	2.83	3.11	5.00	-4.75	4.50	3.75	-3.75	3.75	6.53	6.73	3.73
36.00	CT	fastvelo-slo	28.41	17.58	0.00	1.94	1.50	3.50	-3.75	2.50	3.75	-5.00	2.75	2.63	2.70	4.35
37.00	CT	slowvelo-fa:	22.95	25.50	1.00	3.89	4.61	3.50	0.50	4.25	2.75	2.50	2.50	1.43	3.55	5.60
38.00	GL	slowvelo-fa:	18.85	23.71	1.00	2.33	3.17	3.50	-4.00	2.50	1.00	-0.25	0.75	4.95	3.63	4.55
39.00	CT	slowvelo-fa:	19.83	22.20	3.00	3.33	4.33	0.75	1.25	2.75	1.00	1.25	3.00	6.23	6.23	5.55
40.00	CT	slowvelo-fa:	22.25	16.20	8.00	4.94	3.83	3.75	0.75	4.75	3.75	2.75	4.25	4.65	5.70	6.43
41.00	CT	fastvelo-slo	20.71	20.20	4.00	2.11	1.89	0.75	1.00	0.75	0.25	0.25	0.50	3.05	2.05	2.18
42.00	CT	slowvelo-fa:	21.30	21.50	1.00	2.17	3.33	2.00	-5.00	4.25	0.25	-4.50	0.50	5.69	6.28	5.50
43.00	GL	slowvelo-fa:	30.08	22.64	0.00	1.89	3.28	4.00	-1.75	4.00	3.00	2.00	2.25	1.73	2.25	3.08
44.00	CT	fastvelo-slo	20.48	21.58	1.00	2.89	4.33	1.00	-3.00	0.75	0.00	-2.25	-0.50	5.43	5.88	5.05
45.00	GL	fastvelo-slo	20.31	19.29	1.00	2.78	3.11	0.75	1.50	0.75	2.75	1.75	2.75	3.25	3.93	3.80
46.00	GL	slowvelo-fa:	22.95	18.44	0.00	4.56	2.00	1.50	-5.00	1.75	2.00	-2.50	1.25	3.00	2.90	1.41
47.00	CT	slowvelo-fa:	31.27	23.23	1.00	1.83	3.44	2.50	-2.75	2.50	-0.75	-1.00	-1.00	5.55	5.20	6.56
48.00	GL	fastvelo-slo	19.51	21.45	2.00	3.33	3.00	2.00	-4.00	3.00	3.25	-3.00	4.00	1.58	1.60	1.83
49.00	GL	fastvelo-slo	20.55	19.29	0.00	2.50	3.11	2.50	-3.75	2.75	1.75	0.50	2.00	5.49	4.20	7.15
50.00	CT	slowvelo-fa:	30.72	22.85	0.00	1.56	2.28	2.75	1.25	5.00	3.00	1.25	5.00	2.73	2.68	5.23

id	lpa_vdo_n2	lpa_vdo_n2	lpa_vdo_n2	lpa_vdo_p2	lpa_vdo_p2	lpa_vdo_p2	lpa_vdo_n1	lpa_vdo_n1	lpa_vdo_n1	fastvelo_mi	fastvelo_mi	fastvelo_mi	fastvelo_mi	centered_a	centered_p	centered_e	centered_e	centered_m	centered_b	anx_av_inte	centered_mpain_baseline
1.00	-3.32	-7.91	-0.17	28.29	23.86	20.69	-4.17	-6.08	-7.72	2.05	7.75	-3.17	-1.64	-0.29	2.32	-1.58	-1.53	0.84	-3.97	2.41	0.84
2.00	-44.98	-42.36	-39.92	21.12	17.78	21.31	-13.54	-14.96	-12.00	0.48	2.44	3.54	2.97	0.17	-1.68	-0.53	0.47	0.11	-3.49	-0.25	0.11
3.00										-0.78				-1.71	6.32	0.03	-0.08	-1.99	0.93	0.00	-1.99
4.00	-33.01	-12.05	-20.56	27.22	17.93	23.81				0.25	-8.51	5.89		4.30	1.32	-0.86	-0.30	1.64	-2.46	0.26	1.64
5.00	-7.84	-11.01	-3.68	18.35	23.78	9.46		-1.89	-3.60	-0.33	7.33	-14.33	-1.71	5.49	-1.68	-1.25	-0.53	-1.14	7.37	0.66	-1.14
6.00							-13.51	-9.29	-6.84	-0.23			2.45	0.07	5.32	-1.19	0.03	1.31	6.20	-0.03	1.31
7.00	-26.40	-18.94	-22.29	46.34	39.14	44.02	-15.61	-7.69	-12.47	2.22	-3.35	4.89	-4.78	-0.89	-0.68	0.92	-0.86	-0.54	12.79	-0.79	-0.54
8.00		-2.94	-5.85		15.87	14.10		-6.38	-7.60	-0.10	-2.91	-1.76	-1.22	-4.27	-1.68	1.36	0.53	1.04	-1.32	0.72	1.04
9.00										-0.99				7.69	-1.68	-1.19	-0.14	0.30	-1.87	0.16	0.30
10.00	-15.38	-13.49	-14.13	35.85	35.25	34.71	-0.34	-0.71	-0.42	0.35	-0.64	-0.54	0.29	-1.43	0.32	0.97	0.14	4.26	-3.79	0.14	4.26
11.00	-11.83	-3.66	-8.02	38.40	39.35	28.08	-7.05	-7.90	-8.53	-1.18	-4.36	-11.28	-0.63	-4.47	-1.68	-1.80	-0.92	-0.41	-1.48	1.65	-0.41
12.00	-13.21	-18.76	-8.92	12.01	16.45	12.97	-9.00	-7.42	-1.56	-0.20	9.83	-3.47	5.86	2.27	-1.68	-0.53	-0.53	1.76	-1.21	0.28	1.76
13.00			-2.33			17.66	-7.95	-5.29	-5.11	-0.77			0.18	7.34	1.32	0.92	-1.03	1.63	-3.32	-0.94	1.63
14.00	-11.68	-3.40	-8.21	19.06	8.87	12.25	-11.68	-8.96	-6.48	-0.11	-4.81	3.37	2.49	-2.79	-1.68	-0.42	-0.36	-0.36	0.61	0.15	-0.36
15.00										-0.44				-1.45	1.32	0.47	1.47	0.84	2.03	0.70	0.84
16.00	-37.69	-36.29	-24.63	16.48	20.15	15.67	-12.17	-12.33	-7.85	-0.42	11.66	-4.48	4.48	-0.88	-0.68	0.08	0.20	-1.04	-2.57	0.02	-1.04
17.00	-16.22	-15.56	-16.81	19.30	15.04	16.84	-4.09	-7.53	-3.78	0.10	-1.24	1.80	3.75	6.70	1.32	-0.42	1.31	0.13	-0.82	-0.54	0.13
18.00	-40.58	-36.56	-30.49	28.38	27.29	34.29	-12.12	-9.48	-8.98	-1.30	6.08	7.00	0.50	-2.38	0.32	1.25	0.47	-0.11	2.59	0.59	-0.11
19.00	-34.94	-36.38	-30.76	20.41	24.03	24.43	-5.61	-5.59	-7.06	-0.97	5.62	0.40	-1.48	-2.81	-0.68	2.20	2.53	0.36	-1.69	5.55	0.36
20.00	-13.01		-19.20	32.17		23.96	-12.14		-8.99	0.45				-0.68	-0.68	0.31	0.31	-2.26	-0.20	0.09	-2.26
21.00	-13.15		-17.91	7.17						-0.38				6.81	-1.68	0.97	-0.36	0.16	1.84	-0.35	0.16
22.00	-50.92	-52.56		41.91	34.70		-24.09	-20.27	-15.62	-0.70			4.65	-1.34	-1.68	0.20	-0.08	-0.21	0.66	-0.02	-0.21
23.00								-3.48	-3.51	1.18			-0.03	-2.06	0.32	-0.80	-0.69	0.01	-0.75	0.56	0.01
24.00	-7.83	-4.94	-7.72	19.74	24.48	25.86	-1.69	-5.93		0.79	-2.78	1.38		-1.87	-0.68	1.32	1.25	0.61	-0.61	1.66	0.61
25.00	-18.99	-11.11	-13.16	22.38	11.14	16.60	-7.23	-2.09	-4.88	0.53	-2.05	5.46	-2.79	-1.88	-1.68	0.53	-0.19	-1.55	-1.87	-0.10	-1.55
26.00	-14.97			36.05			-10.51	-9.02	-6.47	-1.20			2.54	-0.53	-0.68	1.44	0.92	-0.49	6.62	1.32	-0.49
27.00	-25.48	-22.93	-24.26	33.71	26.58	25.03	-21.52	-13.82	-12.57	0.40	-1.32	-1.55	1.25	-2.38	-1.68	-1.64	-1.97	0.04	-3.33	3.23	0.04
28.00	-15.99	-4.69	-8.55	31.52	26.90	28.86	-6.11	-3.78	-4.48	0.11	-3.86	1.96	-0.70	-0.01	3.32	0.20	-1.08	-1.11	-2.72	-0.21	-1.11
29.00	-29.00	-31.10	-29.98	36.09	31.27	28.63	-4.83	-7.38	-4.67	-0.02	1.12	-2.65	2.71	-3.12	4.32	-0.92	0.08	-0.24	1.32	-0.08	-0.24
30.00	-9.78	-6.70	-9.89	17.42	9.57	16.85	-6.65	-4.42	-7.37	1.33	-3.19	7.28	-2.95	0.06	-1.68	-0.19	-0.14	-1.86	-1.87	0.03	-1.86
31.00	-4.26	-6.40	-5.92	34.82	27.31	25.81	-16.60	-7.07	-8.73	-0.42	0.48	-1.49	-1.66	0.19	-0.68	-0.03	-0.86	-0.54	-0.39	0.02	-0.54
32.00	-11.62	-14.38	-8.67	21.78	16.55	17.38	-6.95	-5.64	-3.12	1.10	5.71	0.83	2.52	-2.30	0.32	0.53	0.53	1.61	0.79	0.28	1.61
33.00	-1.93	-2.81	0.68	20.10	12.54	15.22	-2.30		-2.83	0.10	3.49	2.68		-4.73	-1.68	1.42	-1.80	0.11	1.75	-2.56	0.11
34.00	-24.98	-14.26	-10.13	22.33	15.63	13.69	-18.50	-7.54	-3.64	-0.48	4.13	-1.95	3.90	6.97	2.32	0.85	0.42	0.16	-0.07	0.36	0.16
35.00	-12.75	-12.28	-6.56	28.64	23.94	14.94	-16.49	-13.59	-8.62	-3.00	5.71	-8.99	4.98	4.67	-1.68	-0.14	0.14	2.34	3.91	-0.02	2.34
36.00	-4.03	-5.47	-3.13	15.90	11.62	8.53	-3.09	-1.31	-2.04	1.65	2.34	-3.09	-0.73	4.50	-1.68	-1.03	-1.47	-1.56	-4.11	1.51	-1.56
37.00	-4.82	-8.94	-14.43	17.70	25.20	28.97	-3.26	-4.40	-5.82	2.05	-5.50	3.77	-1.41	-0.96	-0.68	0.92	1.64	-2.76	3.82	1.51	-2.76
38.00	-5.98	-3.08	-4.69	13.39			-7.54		-2.10	0.92	-1.61			-5.06	-0.68	-0.64	0.20	0.76	2.02	-0.12	0.76
39.00	-20.84	-19.17	-14.33	16.65	17.56	12.06	-10.85	-9.50	-6.07	-0.68	4.84	-5.50	3.43	-4.08	1.32	0.36	1.36	2.04	0.52	0.49	2.04
40.00	-12.10	-16.51	-17.87	39.29	30.49	29.80	-16.26	-12.17	-12.48	0.73	-1.36	-0.69	-0.31	-1.66	6.32	1.97	0.86	0.46	-5.48	1.70	0.46
41.00	-12.35	-6.56	-7.58	14.71	16.65	16.72	-8.13		-3.69	0.13	-1.02	0.07		-3.20	2.32	-0.86	-1.08	-1.14	-1.48	0.93	-1.14
42.00	-16.33	-16.22	-11.74	33.12	26.09	17.85	-6.20	-14.65	-4.97	-0.78	4.48	-8.24	9.68	-2.61	-0.68	-0.80	0.36	1.51	-0.18	-0.29	1.51
43.00	-4.44	-2.88	-10.33	12.90	10.20	17.18	-4.22	-2.94	-4.63	0.83	-7.45	6.97	-1.69	6.17	-1.68	-1.08	0.31	-2.46	0.96	-0.33	-2.46
44.00			-7.37			32.86			-9.48	-0.83				-3.43	-0.68	-0.08	1.36	1.24	-0.11	-0.11	1.24
45.00	-19.72	-16.64	-20.65	38.21	38.04	37.12	-11.24	-8.02	-9.64	-0.13	-4.01	-0.92	-1.63	-3.60	-0.68	-0.19	0.14	-0.94	-2.40	-0.03	-0.94
46.00	-24.89	-16.79	-9.43		9.83	12.18	-4.77	-5.06	-4.92	-1.49	7.36	2.35	0.14	-0.96	-1.68	1.58	-0.97	-1.19	-3.24	-1.54	-1.19
47.00	-3.51		-2.04	4.07		16.73				1.36				7.36	-0.68	-1.14	0.47	1.36	1.54	-0.54	1.36
48.00	-13.66	-3.99	-5.28	29.46	21.26	22.82		-2.91	-5.47	0.23	-1.29	1.56	-2.55	-4.40	0.32	0.36	0.03	-2.61	-0.23	0.01	-2.61
49.00	-15.02	-12.04	-18.63	17.02	25.17	25.74	-8.51	-7.36	-8.55	2.95	-6.60	0.57	-1.19	-3.36	-1.68	-0.47	0.14	1.30	-2.40	-0.07	1.30
50.00	-5.12	-2.52	-4.33	4.53	2.14	9.85	-1.33		-3.98	2.55	-1.80	7.70		6.81	-1.68	-1.42	-0.69	-1.46	1.17	0.98	-1.46
